# What Went Wrong: Corona and the World after the Full Stop

**DOI:** 10.1111/maq.12599

**Published:** 2020-07-21

**Authors:** Carlo Caduff

**Affiliations:** ^1^ Department of Global Health & Social Medicine King's College London

**Keywords:** pandemics, preparedness, power, fear

## Abstract

This article examines the global response to the Covid‐19 pandemic. It argues that we urgently need to look beyond the virus if we want to understand the real seriousness of what is happening today. How did we end up in a space of thinking, acting, and feeling that has normalized extremes and is based on the assumption that biological life is an absolute value separate from politics? The author suggests that today's fear is fueled by mathematical disease modeling, neoliberal health policies, nervous media reporting, and authoritarian longings.

It is as though mankind had divided itself between those who believe in human omnipotence (who think that everything is possible if one knows how to organize masses for it) and those for whom powerlessness has become the major experience of their lives.

——Hannah Arendt

The measures that governments across the world have taken to contain the spread of coronavirus disease are massive and unprecedented. As a result of these measures, life has come to an almost complete standstill, with many countries under lockdown. Never in the history of humanity have such drastic interventions into the lives of populations occurred in the name of health on such a scale and in such a short period of time.

As a result of the world's largest and perhaps most stringent lockdown, millions of daily wage laborers have lost their source of income in India. Health care workers have been attacked and evicted from their homes because they are seen as potential spreaders of contagious disease (Kalra and Ghoshal 2020). Neighborhoods have been scared into panic when an ambulance appears on the street. Due to the sudden ban on any form of transportation, migrant workers have been stranded between the cities where they used to work and the villages where their families are living (Daniyal et al. 2020). Cancer patients have been unable to receive essential medical care because they cannot reach the hospital. It is the poor, the marginalized, and the vulnerable who are most affected by drastic measures, exacerbating already existing inequalities.

In Kenya, the police enforced a coronavirus curfew using teargas and excessive force against presumable violators of lockdown law (Namwaya 2020). In Bangladesh, the government created a special unit to monitor social media and arrest people for spreading “misinformation” about the virus (HRW 2020). In Hungary, parliament passed a law allowing Prime Minister Orbán to limit freedom of speech, defer elections, and suspend rules and regulations by decree (Gebrekidan 2020). In India, state governments released companies from the purview of labor laws, including occupational health laws, to stimulate the economy (Sharma 2020). In Lebanon, the currency collapsed, leaving 75% of the population in need of food aid (Chulov 2020). In the United States, over 33 million people have filed for unemployment benefits (Rushe and Aratani 2020).

Unfortunately, as of the writing of this article, many things remain completely unknown in this pandemic despite intensive investigation. For example, we don't know what helped contain the outbreak in China, and particularly whether government interventions reduced the spread of the virus or if the virus burned out there before moving on to other susceptible populations. The fact is: We simply don't know. Nevertheless, many actors and institutions have proceeded as if they did know, imposing extreme measures that have affected billions of people and that have pushed societies to the edge of collapse by creating poverty, hunger, misery, debt, and unemployment.

Today, many wonder how we ended up where we are. How was it possible for a virus to trigger such a massive response that continues to threaten society and the economy, with so little discussion about the costs and consequences of extreme measures? Why is there widespread agreement that aggressive interventions to “flatten the curve” were necessary and justified? It seems that this unprecedented public health experiment occurred without sufficient consideration of the social, political, and economic consequences.

The failure to consider the impact of extreme measures that have become the norm in many places in the Covid‐19 pandemic has been stunning. The destruction of lives and livelihoods in the name of survival will haunt us for decades.

## How It All Began

The coronavirus disease outbreak seems to have started in the Chinese city of Wuhan in December 2019. In January, the Chinese government put Wuhan and other major cities in the province under lockdown. A lockdown of 56 million people “is unprecedented in public health history, so it is certainly not a recommendation the WHO has made,” Dr. Gauden Galea, the World Health Organization's (WHO) representative in Beijing, emphasized at the time (Reuters 2020). In other provinces, the Chinese government implemented tailored measures, including factory shutdowns and school closures, but not a lockdown or restriction of movement to limit the spread of disease.

Major media outlets in the United States called China's locked‐city strategy deployed in and around Wuhan “harsh,” “extreme,” “severe,” and “controversial,” emphasizing that it offered “no guarantee of success” (Qin et al. 2020). A *New York Times* article noted that “China is trying to halt a coronavirus outbreak using a tactic … with a long and complicated history fraught with social, political and ethical concerns” (Levenson 2020). Experts quoted in the article called the lockdown of cities “an unbelievable undertaking” that would be “patently unconstitutional in the United States.” “That type of thing,” said James Hodge, a professor of law, “is obviously an excessive response.” Another expert cited in the article, historian Howard Markel, pointed to the “darker side of quarantine—its use as a social tool rather than its scientific use as a medical tool.” In the United Kingdom, newspaper articles suggested that the Chinese government would not be able to keep the city of Wuhan “closed for business indefinitely” (Graham‐Harrison 2020).

In February, the virus continued to circulate and soon appeared in other countries. In March, the WHO declared the Covid‐19 outbreak a global pandemic. Despite the criticism of China's approach, a crude and extreme version of lockdown became the international norm promoted by experts, officials, and the media across the world. Concerns with the dark side of quarantine faded rapidly.

A few countries like South Korea veered from this norm and chose instead a classic infectious disease intervention: test–trace–isolate, with a highly centralized approach to public health intelligence gathering. Emphasizing mass testing and meticulous contact tracing to interrupt the chain of transmission, South Korean health officials closed schools and managed the crisis successfully without any lockdowns or roadblocks, and few restrictions of movement. Significantly, South Korea learned from earlier outbreaks of infectious disease (SARS in particular), and imposed central control, used digital technologies, and enforced quarantines, and it witnessed one of the lowest Covid‐19 mortality rates. By the end of April 2020, around 10,000 cases of infection had been detected there, but only 240 people had died.

Germany developed its own testing protocol, which was published on January 17 by the WHO (Beaumont 2020). When the first case was detected on January 28, Germany launched mass testing, systematic contact tracing, and early hospitalization, keeping the mortality rate low and hospitals functional even when cases of infection increased (Mohr and Datan‐Grajewski 2020). Health officials relied on an extensive network of laboratories and were able to conduct over 500,000 SARS‐CoV‐2 (Covid‐19) tests per week (Buck 2020). Along with South Korea, Germany put testing and contact tracing at the heart of the response.

Despite the WHO's emphasis on testing and South Korea's and Germany's early success in reducing the spread of the virus, most countries considered testing at scale as a low priority and relied on an extreme version of the Chinese approach of lockdown. However, in China, the approach was tailored and regionalized; as a WHO report noted, “specific containment measures were adjusted to the provincial, county and even community context, the capacity of the setting and the nature of novel coronavirus transmission there” (WHO 2020). The lockdown focused on the major cities in the most affected province, constraining the life of 56 million people in a country of 1.4 billion. In other words, it was a limited lockdown affecting 4% of China's total population. In contrast to the tailored and regionally differentiated intervention that sought to minimize the socio–economic impact of the response, many other governments across the world imposed nationwide lockdowns that went far beyond China's locked‐city approach. In practice, these lockdowns amounted to curfews (often legalized after the fact by emergency laws).

Italy was the world's first country with a nationwide lockdown/curfew. Many countries followed suit, partly motivated by shocking images of overwhelmed hospitals in Italy's north and partly driven by a disease model report released in the United Kingdom a few days after Italy's surprising national lockdown announcement (Ferguson et al. 2020). This moment of shock and surprise triggered a chain reaction in the pandemic response. The horizon shifted, the inconceivable became possible, and life suddenly felt surreal.

The U.K.’s disease model garnered a lot of attention, creating a sense of urgency that amplified the political pressure because the numbers were alarming (Ferguson 2020). Published by a group of experts without peer review on an institutional website, the report compared Covid‐19 with the great pandemic of 1918, which killed over 50 million people worldwide and suggested, without any evidence, that SARS‐CoV‐2 was “a virus with comparable lethality to H1N1 influenza in 1918.”^1^ Most frightening in all this was not so much the lethality of the SARS‐CoV‐2 virus but the license to rush forward with predictions, abandon basic standards of science, and make dramatic claims to scare people.

For Covid‐19, the report predicted 510,000 deaths in the United Kingdom and 2.2 million deaths in the United States. It presented possible strategies to reduce the impact of the pandemic, but the focus was exclusively on “non‐pharmaceutical interventions.” There was no discussion of testing and contact tracing. A proven public health strategy known to be effective was systematically sidelined in one of the most influential reports to emerge in the Covid‐19 pandemic.

Significantly, the report claimed to focus on “feasibility” of measures and promoted the idea that systematic suppression of transmission would work best—in other words, lockdowns. However, it excluded from any consideration the social, political, and economic implications of lockdowns, noting that “no public health intervention with such disruptive effects on society has been previously attempted for such a long duration of time.”^2^ In addition to ignoring testing and contact tracing as a possible strategy, disregarding the social, political, and economic implications of lockdowns, and conceding that there is no “easy policy decision to be made,” the authors of the report felt confident enough to claim that “suppression” was the “preferred policy option” and the “only viable strategy” that countries across the world needed to implement “imminently.” The lockdowns that were required for suppression would need to be maintained “until a vaccine becomes available” (which they predicted to be “potentially 18 months or more”).

A crude, extreme, and ultimately unsustainable version of the Chinese approach became the international norm. Shutting down society and the economy until a preventive medical treatment becomes available was advanced as an appropriate response and the only possible way of dealing with the crisis, despite the costs and consequences.

Italy figured as an important but fundamentally ambivalent model, shifting the locked‐city into a locked‐country approach. The Italian scenario was sobering and frightening but also inspiring and motivating. To avoid Italy's disaster, governments appropriated the Italian approach of mass confinement and rigid restriction of movement as a one‐size‐fits‐all intervention. In many places throughout the world, including Italy and France, the locked‐country approach took a militarized form with massive deployment of the police to enforce lockdown restrictions.

The locked‐country approach seemed to obviate the necessity of justifying a differentiated strategy that might have looked unequal and unfair and that might have intensified social and political conflicts along multiple internal fractures and fault lines. To avoid the political fallout of a differentiated strategy, which would have required systematic testing, government officials in Europe and elsewhere invoked the politically expedient image of a total threat and suggested that “we are all in the same boat” and that “we are all in this together.”

The idea that regional lockdowns would not be possible and that it was best to treat the virus as a global threat that would uniformly impact all people involved conjuring an image of a united nation confronting a total threat that required everyone's sacrifice. This image relied on a false assumption of equality. Solidarity came to mean not mutual support in a situation of uneven risk, but rather generalization of a sense of danger across a national population perceived as a homogeneous body under attack. The ideology of national pandemic solidarity—putting everybody under confinement and treating everyone the same—obscured the reality that lockdowns mean different things to different people, and that not everyone is equally exposed or equally vulnerable. Both the virus and the lockdown disproportionately affected those who were already vulnerable along lines of age, class, and race.

Dramatic references to the magnitude of the threat served as justification for nationwide lockdown policies. This extreme and unprecedented blanket approach systematically imposed on entire populations was driven by a number of factors that variously prevailed in different countries across the world: a growing sense of panic, constant media sensationalism, deep authoritarian longings, increasing political pressure to contain the spread of the virus, disturbing accounts of overwhelmed hospitals unable to cope with the surge of patients, misleading mortality calculations, and, most importantly, a trust in the power of mathematical disease modeling.

Throughout the Covid‐19 pandemic, there has been an abiding assumption among observers and the public that it is clear what is happening; that everyone knows what is going on because everyone can see it on television. However, what an endless stream of media reports from around the world have continued to obscure is the fact that it is impossible to know what is happening in a population when there is no systematic testing. The lack of testing created a void that was filled by the flexible evidence of disease modeling. In the absence of robust data, disease modeling emerged as the presumably best and only available science to inform policy.

Media hyperbole focused on absolute numbers independent of context and made Covid‐19 deaths politically visible. Flexible disease modeling (often based on data derived from viruses such as influenza) took the place of accurate epidemiological surveillance. Scientific papers published online without peer review made scary projections and painted a grim picture. Widely reported simulation models created shock effects that shaped government policies.

A narrow focus on numbers played an important role in understandings of the magnitude of the threat, fueling fear and panic in the absence of actual evidence. A distinct imaginary took hold, “the imaginary of an unprecedented event,” which seemed to require an unprecedented response (Kelly 2018). There was a widespread sense, among experts and the media, that the SARS‐CoV‐2 virus was much more lethal than influenza. That this pandemic was different from influenza and thus necessitated a different approach was typically claimed on the basis of the case‐fatality rate, the number of deaths as a subset of those infected with SARS‐CoV‐2. The case‐fatality rate played a crucial role in the justification of the public health experiment now unfolding before our eyes.

## Obscuring the Lack of Evidence with Numbers

Estimates of the case‐fatality rate initially varied hugely from 0.17% to 17%. In an article published in *The Lancet*, scientists claimed the case‐fatality rate could even be as high as 20% (Baud et al. 2020). In early March 2020, the WHO director‐general stated that the case‐fatality rate for SARS‐CoV‐2 was 3.4%. He added: “By comparison, seasonal flu generally kills far fewer than 1% among those infected.”

Whatever the estimates, the fact remains that it is impossible to calculate the case‐fatality rate in the absence of systematic testing. Given the lack of evidence, the only scientifically valid statement at the time would have been to say that we simply don't know how lethal the virus is.

Early on in this pandemic, it became clear that over 40% of infected people were experiencing no symptoms at all at the time of testing (Gudbjartsson et al. 2020). This means that a surveillance regime where only people with symptoms were tested will automatically exclude a large number of infections. Additionally, patients with symptoms are much more likely to die than asymptomatic people. The result is an exaggerated case‐fatality rate.

Testing strategies differed across countries and changed within countries over time. For example, on February 25, 2020, the Italian Ministry of Health published a revised policy for testing, prioritizing patients with severe clinical symptoms (and thus higher chances of dying). This change in policy resulted in an apparent increase in the case‐fatality rate of 3.1% on February 24 to 7.2% on March 17 (Onder et al. 2020). Suddenly, the virus seemed to have become much more deadly. However, this increase was a numerical illusion—a statistical artifact. There was no change in the lethality of the virus.

Changes in testing policy occurred in many countries and even across regions where different tactics for counting deaths were used. In China, test‐positive asymptomatic patients were excluded from being counted as cases of infection (Wu et al. 2020). In Belgium, deaths were counted independent of any testing (Schultz 2020). Of 52% of all deaths, only 4.5% turned out to be confirmed by laboratory test as Covid‐19 positive. Almost half of all victims were merely suspected to be linked with the virus but had never actually been tested.

There was and remains no agreement among experts and officials on what counts as a death caused by the virus. In Italy, Covid‐19‐related deaths were defined as those occurring in test‐positive patients, “independently from preexisting diseases that may have caused death” (Onder et al. 2020). This is particularly concerning in terms of data quality because the vast majority of deaths occur in patients who are older than 65 with one or more comorbidity. Test‐positive patients who die because of heart disease or terminal cancer are not necessarily dying because of SARS‐CoV‐2 infection. Yet they appear in the statistics of some countries. This confusion between patients who die with the virus and those who die from it has had an impact on the data and their quality, making comparisons between countries impossible.

Further, almost all tests that are done use RNA tests, which can detect an infection only as long as the virus is present in the body. These tests, however, cannot tell whether a person had the virus in the past. Only serological tests for antibodies against the virus can provide an accurate picture of how many people have been infected in a given population. And yet, such systematic serological studies were and are missing.

Given the lack of testing and taking into account the role of selection bias, the large number of asymptomatic cases, the confusions in case definitions, the changes in testing policies, and the difficulty of knowing who is dying *with* versus dying *from* the disease, the denominator for calculating actual death rates cannot be reliably determined. Without a denominator, it is mathematically impossible to calculate the case‐fatality rate. Nevertheless, despite the lack of data, experts, officials, and the media have remained transfixed by the assumption of clarity and reliability of numbers, and they continued to circulate wild estimates, unleashing a pandemic of scary charts with exponential curves.

Over the last weeks of March, more and more testing was done globally, and more testing continues as of the time of this publication. Not surprisingly, estimates of the case‐fatality rate have come down significantly, because the denominator has gone up due to the increase of testing. In Iceland, 12% of the population has been tested using RT‐PCR‐based tests independent of symptoms, suggesting a case‐fatality rate of 0.55%.^3^, ^4^ This figure is six times lower than WHO's official estimate for Covid‐19.

The Center for Evidence‐Based Medicine at the University of Oxford noted that if one assumes that 1% of Iceland's population is infected, then the corresponding infection‐fatality rate would be 0.03%.^5^ A study using both RT‐PCR‐based and serological tests conducted in one of Germany's most affected regions indicated a case‐fatality rate of 0.37% and an infection‐fatality rate of 0.06% (Streeck et al. 2020).

We know from epidemics and pandemics of the past that the case‐fatality rate is often massively overestimated at the beginning of an outbreak because case detection is limited, largely based on hospital patients and typically biased toward the severest cases of disease. When the H1N1 swine flu pandemic occurred in 2009, the estimated case‐fatality rate varied between 0.1% to 5.1% in the first 10 weeks of the outbreak. In 2019, a decade after the pandemic, the WHO reported that the swine flu pandemic turned out to have a case‐fatality rate of 0.02%.^6^ This means that the actual case‐fatality rate was five times lower than the lowest estimate.

Social science scholarship has shown how numbers can deceive. Numbers have the ability to reveal as well as conceal. Therein lies their magic. They appear as seemingly neutral bearers of truth. They offer a sense of mathematical precision, making things seem more certain than they actually are and displacing attention away from the conditions under which they were produced. Abstracting from limitations on the conditions of their production and treating numbers as absolute is dangerous because it makes things comparable that are not comparable, because it suggests scientific knowledge where there is lack of evidence, and because it creates the sense of a major threat obscuring the differential nature of risk. What using numbers this way fails to account for is the fact that not everyone is at risk in the same way.

## Structural Fragilities

Among the more interesting figures of the SARS‐CoV‐2 pandemic is the number of deaths per million inhabitants per country. This number is probably more reliable than the case‐fatality rate because deaths are less likely to be missed (ignoring for now the case of Belgium and the difficulty of defining deaths caused by SARS‐CoV‐2) and because the denominator, a country's population, is known.^7^ Here are the current numbers of deaths per million inhabitants for five countries as of May 15, 2020:

Spain: 584

Italy: 519

France: 420

Germany: 95

South Korea: 5

The staggering differences between countries cannot solely be explained by demography or rates of infection (some countries seem to have more infected people per million inhabitants than others and so might be overwhelmed, though this is also a question of time—how many cases per week per region). What the differences might reveal (and it is important to note that they may well change) is that some health care systems are able to deal with the crisis in a better way than others. The structural fragilities of an underfunded, understaffed, overstretched, and increasingly privatized and fractured health care system contribute to higher mortality rates (Adams 2020). In a sense, each society has the mortality it deserves (Canguilhem 1989: 161).

Where medical care is easily accessible, with sufficient and well‐trained staff, and with capacity flexibility, patients are more likely to receive better care and survive. In this sense, it matters that Spain turns out to have 3 beds per 1,000 inhabitants, Italy 3.2, France 6, Germany 8, and South Korea 12.3.^8^ Although beds per inhabitants is a crude indicator, it is noteworthy that Germany can rely on over 30,000 staffed intensive care beds, out of which only 11,500 were occupied in early April 2020 (see Mohr and Datan‐Grajewski 2020). This at a time when there were more cases in Germany than in France and the United Kingdom and slightly less than in Spain and Italy. Germany's was clearly not a health care system overwhelmed by a sudden surge of patients. Ironically, organizations such as the OECD frequently scolded Germany's health care system in the past for “oversupply” of hospital beds and its “inability” to “rationalize hospital capacity” (Kumar and Schoenstein 2013).

This means that the case‐fatality rate is not just dependent on the biological nature of the virus and the age and health profile of the population (people most at risk of death are older than 65 with one or more comorbidity). The case‐fatality rate also depends on systematic testing, meticulous contact tracing, well‐trained health care workers, nursing homes with adequate resources, and the ability of the health care system to cope with the crisis (excess as well as surge capacity) and provide high‐quality medical care, particularly keeping medical workers safe and healthy. In this sense, the pandemic has and will continue to brutally expose policy failures and structural health care system deficits.

The situation in many hospitals in Italy, Spain, and France is troubling, especially in densely populated areas. But it is important to understand why some of these highly visible institutions of care were overwhelmed. Lombardy, Italy's most affected region, has long been an experimental site for health care privatization: community‐centered care “has been all but wiped out” (Bagnato 2020). The lack of general practitioners and the defunding and low emphasis on community care have increased the pressure on hospitals in urban centers. These hospitals have neither excess nor surge capacity to cope with a sudden rise in demand.

Over the past five years, hospitals across Europe held numerous strike actions “with doctors and health workers complaining of funding cuts, a government reduction in the number of beds and a serious lack of medical staff leading to dire working conditions for emergency room staff” (Chrisafis 2019). Hospital systems in Italy, Spain, and France were on the brink of collapse even before the virus arrived.

The most telling demonstration of the structural contradictions of pandemic preparedness under neoliberalism occurred, not surprisingly, in the United States. As American newspaper articles reported, hospitals across the country deferred regular medical services to free up space, equipment, and staff for the pandemic response. When patients started to avoid hospitals due to fear of infection, a main source of income was drastically cut off, “causing huge losses that have forced some hospitals to let go of health care workers as they struggle to treat infected patients” (Harris and Schneider 2020). Facing a “financial nightmare,” hospitals filled their intensive care units with patients who did not really need intensive care so that they could charge more and make up for the financial loss. Additionally, administrators cut salaries, laid off hundreds of staff, and sent others on unpaid leave, weakening the health care system further in the midst of the pandemic response.

When a new virus appears, things start to fall apart. Once everyone gets scared, extreme measures are implemented, in a more or less improvised manner, and trillions of dollars, euros, and pounds are pumped into the economy to make up for the loss. Once the worst is over, however, the normal crisis continues, and the structural fragilities remain (Caduff 2015).

## Rebuilding the World after the Full Stop

This pandemic will haunt us all for decades in ways that we can barely imagine at this point. The nature and sheer scale of the interventions that we have witnessed are staggering, and the consequences—social, political, and economic—remain unforeseeable. There are no systematic accounts of the implications and repercussions seen so far, nor do we have any idea about the number of indirect deaths due to the lockdowns/curfews, the social distancing and the self‐isolation. We have yet to see a realistic plan that would outline how we might learn to live with a virus that is unlikely to disappear any time soon (Sullivan and Chalkidou 2020).

In the meantime, I suggest that we reframe the corona conversation to cut through the confusion and dimness that is pervading this pandemic in the following ways:

### I. Orientations

1 The emergence of new viruses in human populations is normal. It has happened before; it will happen again.
2 Coronaviruses are common and circulate widely in humans. They have infected people and killed thousands year after year, especially in winter.
3 Worldwide, between 300,000 to 500,000 people die from influenza viruses every year. The SARS‐CoV‐2 virus has killed 300,000 people so far. There is no doubt, SARS‐CoV‐2 is causing a serious infectious disease, but so far it is still in the range of what we observe in terms of mortality during a severe influenza season. The main difference is the speed of infection, the clinical picture of the disease, and the impact on demographically older populations causing massive compression of morbidity and mortality that is overwhelming weak health care systems with no excess and little surge capacity.
4 The 1957 influenza pandemic killed between 1 and 2 million people worldwide, and the 1968 influenza pandemic killed between 2 and 4 million people. As of the writing of this article, Covid‐19 has killed 300,000 people, according to the official numbers. Clearly, the world has witnessed worse pandemics, including 1.3 million deaths due to TB each year, 770,000 deaths due to HIV infections each year, and 435,000 deaths due to malaria, all preventable and treatable conditions.
5 This observation does not mean that influenza and Covid‐19 are clinically similar or that nothing should be done to contain the spread of SARS‐CoV‐2 and mitigate the consequences. However, it raises the question of why fear and panic are spreading like wildfire, provoking such extreme measures, and why experts and government officials are willing to mount an unprecedented effort for SARS‐CoV‐2 but have never considered similar interventions for the 300,000–500,000 people who die every year due to influenza. Influenza is a relatively well‐known virus. To say that SARS‐CoV‐2 is an unknown virus doesn't automatically justify the most extreme measures that the world has ever seen.

### II. Diagnosis

1 What makes this pandemic unprecedented is not the virus but the response to it.
2 Extreme measures to contain the spread of the virus have resulted in extreme fallouts. It is difficult to overestimate what we are witnessing today.
3 The pandemic response has pushed the world into a space of fragility and uncertainty. There hovers a “perhaps” over everything now (Caduff 2015). Blinded by the urgency of the immediate moment, the response has created an opening for actors and institutions to push agendas and reorder the world. We will grapple for years to come with the changes that are happening today.
4 The response to the disease is driven by a fantasy of control that overestimates and overreacts. This fantasy has caused and is causing enormous harm. It is unrealistic, misleading, and bound to fail. A pandemic like this cannot be controlled; it can only be managed.
5 If we keep using words such as control, we are only setting ourselves up for disappointment. This pandemic is far from having found a language that is adequate to the problems it is posing. We urgently need new concepts but seem to have little imagination.
6 The urgency of the crisis has displaced reliance on basic standards for quality and control of quality of scientific research. Papers are published without peer review. Claims are made without evidence. Perhaps not surprisingly, given the fragile health care infrastructures in some countries, speed appears to be more important than quality, rigor, and integrity.
7 Underscrutinized science, lack of data, speculative evidence, strong opinions, deliberate misinformation, exaggerated mortality rates, the 24/7 news media attention, and the rapid spread of dramatic stories on social media have led to poor political choices and major public anxiety.
8 We are afraid of Covid‐19. We are not afraid of influenza. We see one thing as a public health emergency and another as a fact of life. Today, we are learning an old insight the hard way: Not every life and not every death are equal. Some deaths are more important than others, drawing more attention, triggering a bigger response and mobilizing more resources.
9 In the Covid‐19 pandemic, the belief seems to have taken root that health is an absolute value and that every life needs to be saved by all means. Meanwhile, millions of people are dying of influenza, TB, HIV, malaria, and diarrhea, not to mention chronic diseases and accidents. There seems to be less political urgency for these preventable deaths.
10 Some health care systems were overwhelmed in this pandemic. Others were not.
11 For decades, governments have underfunded, understaffed, and privatized health care systems across the world, and these trends have exacerbated the impact of the pandemic.
12 The response to SARS‐CoV‐2 took a particular shape, converging in extreme measures that have become the norm in many countries. Questions that remain include: Was it the only possible way of managing the crisis? Why has a crude version of China's approach become the dominant model? At the heart of this pandemic was and is the widespread assumption that there were and are no alternatives to extreme measures implemented on entire populations with little consideration of cost and consequences. This is not true. As some countries have shown, adequate testing and less drastic policies of social distancing work well to manage the pandemic.
13 It seems that some officials saw Covid‐19 as a disease that could be contained. As the WHO director‐general suggested in early March 2020, “we don't even talk about containment for seasonal flu—it's just not possible. But it is possible for Covid‐19.” This perception may have contributed to the radically different approach seen across many countries.
14 The idea of “flattening the curve” is often seen as the optimal solution, but there is no guarantee that the effort to do this will actually impact the total number of deaths over the long run of the disease's presence in any community. It may ultimately simply spread the same number of deaths over a longer period of time and thus perhaps reduce the pressure on hospitals but not overall mortality.
15 Nationwide lockdowns are not a solution. They prevent infection as long as they are in place, but they also keep people susceptible. This is particularly concerning in a pandemic where the virus has become endemic. Once lockdowns are lifted, the number of infected people may well rise again later. This is why it has been so hard for countries who adopted this strategy to return to normal life—the strategy is not sustainable over the long run.
16 As Andrea Bagnato noted about the stay‐at‐home strategy: “It is not in the harshness of its lockdown, but in the effectivity of separating the infected from the non‐infected, that China's response has excelled: a centralized system of dedicated structures (called Fangcang) was built in no time, where all patients and their contacts were treated and divided in four groups according to severity. Instead, Lombardy simply closed everything down. And it becomes clearer by the day that the main landscapes of infection were not public spaces, but hospitals, retirement homes, workplaces, and indeed private homes” (Bagnato 2020).
17 In Germany, 86% of the people who died due to Covid‐19 are 70 years or older (Mohr and Datan‐Grajewski 2020). A majority of the patients who died have one or more underlying health condition such as hypertension, diabetes, cardiovascular disease, chronic respiratory disease, or cancer. This means that the pandemic is killing predominantly people with an already reduced life expectancy. The key question then becomes excess deaths—the difference between the statistically expected number of deaths and actually occurring deaths over a period of time. There is no doubt that there will be excess deaths due to Covid‐19, but it is unclear how large that number will be.
18 The pandemic response has produced a substantial rise in the number of people who now live with untreated illness. Prohibition of public transport has made it difficult for patients and staff to reach hospitals. Patients with conditions other than Covid‐19 avoid doctors because they are afraid of getting infected. Emergency Room attendance dropped substantively the world over. Cancer referrals decreased and cancer screening services stopped entirely. Rural health services in countries such as India crashed. Essential public health programs have been paused; many resources have been reallocated. This means that patients are neglected, receiving no or less medical care, leading to untreated illness and a rise in mortality.
19 A virus causes disease, not hunger. It is not the pandemic, but the response to it that threatens the livelihood of millions of people. In many countries, both rich and poor, the trends are shocking. In India, children die of starvation. Farmers commit suicide because they are unable to harvest crops. Stranded daily wage laborers drop dead after walking hundreds of miles. The poor, marginalized, and vulnerable bear the brunt of the pandemic response.
20 The lockdown is a political mechanism not simply for the prevention but for the redistribution of negative effects. Lockdowns shift negative effects away from hotspots of public attention to places where they are less visible and presumably less serious. In this way, they are part and parcel of a politics of inequality.
21 This pandemic is not just about health, it is about fear, and the objects that are singled out and then made the ground and motivation of systematic thought and action.^9^ To be afraid has become an obligation, a responsibility, a duty. People are afraid not just because of what they experience but because they are told to be afraid and encouraged to inhabit the world with fear of “foreign bodies” and “invisible enemies.”
22 Public discourse is highly moralized. Looking for someone to blame, individuals are exposed as “super‐spreaders” responsible for the rising number of cases. On social media, “lockdown warriors” accuse citizens of lack of patriotism and failure to “do their duty” in the face of danger. In this highly moralized public discourse, life is considered an absolute value that can justify almost every form of disciplinary intervention in the name of health.

### III. Toward Another Politics of Life

1 Public health needs to be front and center in any infectious disease intervention. Investing in strong public health infrastructures should happen even when there is no pandemic.
2 Mathematical disease modeling cannot replace systematic epidemiological surveillance on the ground. The most effective way to manage an infectious disease outbreak is to test, trace, and isolate.
3 Interventions need to be phased over time; they need to be dynamic, regionally targeted and risk based. All interventions must take into account the social, political, and economic impact, as well as the indirect impact on other health conditions. Interventions that do this will create management strategies that work to minimize collateral damage.
4 Absolute numbers cannot be used for policy, they only fuel fear and panic.
5 National lockdowns are not a solution. They protect people temporarily, but they also leave them susceptible. Once restrictions are lifted, cases of infection are likely to increase again. There is no exit from the pandemic; there is only an exit from the response to it.
6 We are still at an early stage of understanding how best to clinically manage Covid‐19 both as a disease and as a risk factor to potentially vulnerable populations. It is vital to find better ways of sharing quality data and effective practice to ensure health systems learn and adapt quickly.
7 What this pandemic shows is a lack of preparedness. This will come as a surprise, given the billions of dollars, euros, and pounds that were spent over the last 15 years on pandemic preparedness, including experience with past epidemics and pandemics such as Ebola and swine flu. How can it be that hospitals ran out of N95 masks in week one? Where did all the billions spent on preparedness go? Outsourced production capacity and insufficient stockpiles of personal protective equipment put nursing home residents, community health care workers, and hospital staff at risk, weakening health care systems further.
8 Key preparedness concepts need to be at the heart of the response. Fifteen years of pandemic preparedness seem to have evaporated into thin air in this pandemic. Instead of activating existing plans and drawing on concepts such as the Pandemic Severity Assessment Framework, countries imposed a massive, untested, and unproven generic lockdown with unforeseeable social, political, and economic repercussions.^10^
9 SARS‐CoV‐2 is less lethal than every single scenario exercise that has been conducted for preparedness planning by governments and non‐governmental organizations in Europe and America. It will be important to understand why key preparedness concepts were sidelined in this pandemic, despite the attention that preparedness received and the substantial resources it consumed for over a decade.
10 The fear of death is powerful in societies eager to repress the inescapable reality of death. In such a context, it is important to flatten the curve of extreme speaking, feeling, and acting. What was and will always be urgently needed is moderation and perspective.
11 To continue to engage in today's competition for ever more extreme predictions is dangerous. It will only support those who ignored the virus initially and who are more than willing to blame it now for the mess. Equally dangerous is a public health populism of clapping hands that leaves out any consideration of the social, political, and economic costs and consequences of sweeping interventions.
12 Attempts to obscure political failures are growing rapidly. Those who contribute to extreme predictions and apocalyptic readings of the current situation are only contributing to the obfuscation of the policy failures and underlying structural issues that are responsible for many of today's problems. There are already attempts in countries such as the United Kingdom and the United States to rewrite failure as success. Not surprisingly, governments are calling on citizens to participate in public performances, demonstrate national unity in the face of danger, and celebrate collective strength and resolve. Fighter jets soaring through the sky and helicopters showering rose petals on “frontline warriors” are militarized state spectacles. But health care workers deserve more than patriotic feelings and symbolic gestures; they deserve better health care policies. To challenge and critique now is essential.
13 The story of how the Chinese approach became a model for generic lockdowns in the Global North and then exported to countries in the Global South is important to note, particularly considering the dramatic consequences for millions of people struggling to survive without any source of income. Ironically, these extremely restrictive lockdowns were sometimes demanded by people eager to criticize the authoritarianism of the Chinese state. Across the world, the pandemic unleashed authoritarian longings in democratic societies, allowing governments to seize the opportunity, create states of exception and push political agendas. Commentators have presented the pandemic as a chance for the West to learn authoritarianism from the East. This pandemic risks teaching people to love power and call for its meticulous application.
14 Pandemic time is an auspicious time for all kinds of political projects.
15 As a result of the unforeseeable social, political, and economic consequences of today's sweeping measures, governments across the world have launched record stimulus bills costing trillions of dollars, pounds, pesos, rand, and rupees. Earmarked predominantly for individuals and businesses, these historic emergency relief bills are pumping staggering amounts of money into the economy, but, ironically, they are not intended to strengthen the public health infrastructure or improve medical care. The trillions that governments are spending now as stimulus packages surpass even those of the 2008 financial crisis and will need to be paid for somehow. Today, there is a massive global recession in the making. If austerity policies of the past are at the root of the current crisis with overwhelmed health care systems in some countries, the rapidly rising public debt is creating the perfect conditions for more austerity in the future. The pandemic response will have major implications for the public funding of education, welfare, social security, environment, and health in the future.
16 If you think something good will come out of this crisis, you should think again. Today we are just driving faster and with a much bigger car, but it is the same road with the same destination.

## A Difficult Place for Critique

Wolf Bukowski notes that the political discussion in Italy is now dominated by an “uncritical ‘responsibility’” that cannot find a place outside the imperative to contain the virus. “The right intuition that ‘we should not question the reality of the epidemic’ shifts all too easily into ‘we should not question the government's response to the epidemic’” (Bukowski 2020). In such a context, any control intervention imposed by the state is perceived as lawful, and no democratic discussion and debate appears necessary (“Let the experts speak!”).

In other places, critique has become difficult for other reasons. The tragedy of today's political moment in the United States, the United Kingdom, and Brazil is that right‐wing politicians pushed many into embracing measures that one thought were only possible in authoritarian regimes. Here, an engagement in critical analysis has become almost impossible because it is seen as playing into the hands of Trump, Johnson, and Bolsonaro, political figures who seem unconcerned with public health and the staggering inequalities that afflict our world and whose public statements have reached an unmatched level of ignorance and incompetence. However, it is important to understand that the strategic combination of confusion, contradiction, and the play of extreme opposites is foundational for authoritarian rule. Everything that instills a sense of disorder and that intensifies the crisis magnifies the desire for decisive action.

In this article, I have tried to carve a path through the morass of fear, panic, and desire for control to see how one can sustain a critical analysis of the pandemic response. As scholars and citizens, we have the obligation to think beyond the crisis, create openings in the world, and consider, critically and democratically, how we want to govern ourselves. As Veena Das underscores, it is important that we do not let our “love for the subtle and nuanced understanding of issues disappear on the grounds of needs for the rough and the ready in an emergency” (Das 2020). The pandemic and the response to it will require us to reimagine lives, rebuild conditions of existence, and find better ways of doing science and politics. Like every engagement in a serious pedagogical project, it will entail a reconsideration of the objects we desire.

Today's fear is fueled by four main forces:

1 Mathematical disease modeling—a flexible and highly adaptable tool for prediction, mixing calculations with speculations, often based on codes that are kept secret and assumptions that are difficult to scrutinize from the outside.
2 Neoliberal policies—systematic disinvestments in public health and medical care that have created fragile systems unable to cope with the crisis.
3 Nervous media reporting—an endless stream of information, obsessed with absolute numbers, exploiting the lack of trust in the health care infrastructure and magnifying the fear of collapsing systems.
4 Authoritarian longings—a deep desire for sovereign rule, which derives pleasure from destruction and tries to push the world to the edge of collapse so that it can be rebuilt from scratch.

This set of forces inspires thought, action, and passion in powerful ways. Energized by the thrilling experience of witnessing “history in the making,” actors and institutions have seized the opportunity to reorder the world, push political agendas in the name of survival, and shape life for years to come. The pandemic has become an auspicious moment to change the rules of engagement and expand the scope of scientific, medical, and political authority over bodies and populations. It is an occasion to publish papers and make dramatic statements, to feel relevant and important to the world, and enjoy the moment in the limelight. In the midst of death and destruction, the pandemic creates opportunities for innovation, domination, and profit‐making.

This unexpected opening connects elites in science, politics, and the media, releasing shocks of information, instruction, and command that are pushing hard against our confined, anxious, restless bodies. Mathematical disease modeling, neoliberal policies, nervous media, and authoritarian longings fuel a fatal spiral centered around the fear of collapse.

This fear is now literally in the air; it moves in and out of us with every breath; it operates as animating medium of our intense isolation and immobility. Pandemic fear is unnerving and mentally exhausting. Yet for those who embrace the feeling, it has the power of sustaining a state of excitement—excitement derived from the secret pleasure of spoiling a precious thing, wasting enormous resources, and engaging in an all‐consuming project with total dedication. What we might call the provocation of the crisis—its intensification, expansion, and totalization beyond any notion of utility—seems so excessive and extreme that it borders on sheer madness. What could be more dangerous, more daring, more exciting than a walk on the wild side, an excursion to the other side of reason?

## Coda

Melodramatic phrases such as “beating the virus,” “winning the war,” and “defeating the darkness” are rhetorically powerful and contagious. Equally popular notions like “corona heroes” and “lockdown warriors” are symptoms of overidentification in a hegemonic discourse of power. All these terms reveal how this pandemic is “fabulously textual, through and through,” and, at the same time, is lacking a source of symbolization strong, creative, and disturbing enough to move our engagement with the world beyond the most conventional of tropes (Derrida 1984). The language that we are asked to adopt today, in the midst of this outbreak, is contaminated with words that are stiff, stale, and corrupt like putrid air.

Given that so much of today's response is based on and driven by mathematical disease modeling and that millions of lives and livelihoods are being destroyed before our eyes, it is not an option anymore to exclude the “externalities” of a pandemic response that lacks imagination and that has resorted to the crudest interventions of all: the full stop. For those with permanent jobs, a comfortable couch, and no daycare duties, this unforeseen interruption may feel like a gift, a welcome relief from the non‐stop world of global capitalism. But for millions of people living in less privileged parts of the planet, the pause button spells unemployment and hunger, not breaktime and downtime. Without income, food, and access to basic health care, people are not making the most of the confinement outside in the garden; they are desperate and dying.

We urgently need to look beyond the virus if we want to understand the real seriousness of what is happening today. How did we end up in this strange space of thinking, acting, and feeling that has normalized extremes and that is based on the assumption that biological life is an absolute value separate from politics? Never has it been more important to insist that another politics of life is possible.

The latest Imperial College disease model report summarizes the staggering blindness that has prevailed in this pandemic: “We do not consider the wider social and economic costs of suppression, which will be high” (Walker et al. 2020). The time to suppress the costs of suppression and cast the consequences of interventions as an externality to model‐based policy is over.

## Notes

1 “The global impact of COVID‐19 has been profound, and the public health threat it represents is the most serious seen in a respiratory virus since the 1918 H1N1 influenza pandemic.” “The last time the world responded to a global emerging disease epidemic of the scale of the current COVID‐19 pandemic with no access to vaccines was the 1918–19 H1N1 influenza pandemic.” These claims are utterly misleading, ignoring the influenza pandemics of 1957 and 1968, including the HIV/AIDS pandemic, Ebola, and many other infectious disease outbreaks that have killed millions of people worldwide with no vaccine available. Last but not least, the toll of Covid‐19 is not even close to the toll of the 1918 influenza pandemic.

2 The report distinguishes between two strategies, “mitigation” and “suppression.” The latter corresponds broadly with the lockdown strategy.

3
https://www.who.int/dg/speeches/detail/who-director-general-s-opening-remarks-at-the-media-briefing-on-covid-19—3-march-2020

4
https://www.worldometers.info/coronavirus/ Accessed 5/15/2020.

5
https://www.cebm.net/covid-19/global-covid-19-case-fatality-rates/

6
https://onlinelibrary.wiley.com/doi/full/10.1111/irv.12074

7
https://www.worldometers.info/coronavirus

8
https://data.oecd.org/healtheqt/hospital-beds.htm

9 “These days I sometimes catch myself wishing to get the virus—in this way, at least the debilitating uncertainty would be over… A clear sign of how my anxiety is growing is how I relate to sleep. Till around a week ago I was eagerly awaiting the evening: finally, I can escape into sleep and forget about the fears of my daily life… Now it's almost the opposite: I am afraid to fall asleep since nightmares haunt me in my dreams and awaken me in panic—nightmares about the reality that awaits me” (Slavoj Žižek 2020). Is Barbarism with a Human Face our Fate? Critical Inquiry blog. Accessed 27.4.2020. https://critinq.wordpress.com/2020/03/18/is-barbarism-with-a-human-face-our-fate/

10 CDC 2007. Interim Pre‐pandemic Planning Guidance: Community Strategy for Pandemic Influenza Mitigation in the United States.
